# Artificial Intelligence for the Detection of Hypertrophic Cardiomyopathy From Standard Electrocardiogram

**DOI:** 10.1016/j.jacadv.2026.102914

**Published:** 2026-06-16

**Authors:** Jakob Park, Jonathan Kermanshahchi, Christopher J. Love, Klevin Roger Reyes, Alexander Kukuev, Sarit Meshesha, Katia Kon, Florian Rader

**Affiliations:** a Smidt Heart Institute, Department of Cardiology, Cedars-Sinai Medical Center, Los Angeles, California, USA; b Viz.ai Inc, San Francisco, California, USA; c California University of Science and Medicine, Colton, California, USA

**Keywords:** artificial intelligence, electrocardiogram, hypertrophic cardiomyopathy, screening

## Abstract

**Background:**

Hypertrophic cardiomyopathy (HCM) is often diagnosed late, increasing avoidable risk and delaying treatment. Artificial intelligence (AI) for 12-lead electrocardiograms (ECGs) may identify undetected HCM earlier.

**Objectives:**

This study aimed to evaluate the performance of an AI algorithm for HCM detection and identify predictors of correct classification.

**Methods:**

Of 314 patients with cardiac magnetic resonance imaging (cMRI) for suspected HCM, 150 had analyzable ECGs and confirmed HCM by physician review of medical records and cMRI (ground truth). Eighty-three control patients without cardiomyopathy were included. A proprietary algorithm, Viz HCM (Viz.ai, Inc), labeled ECGs as HCM-positive or HCM-negative; the ECG closest to each patient’s cMRI date was compared to ground truth. Diagnostic performance was evaluated by the area under the curve of the receiver-operating characteristic curve and at the prespecified threshold. Predictors of correct detection were determined by multivariable logistic regression for age, sex, race, maximal wall thickness, and hypertrophy subtype.

**Results:**

The mean age of all 233 patients was 56 years, and 62% were male. The algorithm identified HCM with an area under the curve of 0.946 (95% CI: 0.916-0.970), sensitivity of 58% (95% CI: 50.0%-65.6%), and specificity of 100% (95% CI: 95.6%-100%). Apical subtype was a significant predictor of correct detection (adjusted OR: 4.71; 95% CI: 1.71-15.48; *P* = 0.005). In 9 of 28 patients with ECGs available at least 1 year prior to cMRI, the algorithm detected HCM 2.6 years (median) before clinical diagnosis.

**Conclusions:**

The AI ECG algorithm demonstrated highly specific HCM detection confirmed by cMRI and may improve early identification of unrecognized HCM.

Hypertrophic cardiomyopathy (HCM) is the most common inherited cardiomyopathy, prevalent in the community with an estimated occurrence of 0.2% (1 in 500) and up to 1.4% in the general adult population and having an estimated global disease burden of up to 20 million patients.^1^^,^^2^ Despite this prevalence, HCM remains underdiagnosed due to nonspecific (or a lack of) HCM-related symptoms. Reports have described a diagnostic time delay of up to 2 years,^3^ as well as a potential diagnostic gap of undiagnosed patients of up to 85% in the United States alone.^4^ Patients with HCM have a higher morbidity and mortality compared to the general population with a higher clinical and economic burden on the health care system^3^^,^^5^ which early diagnosis could help mitigate by allowing for timely initiation of targeted and preventative treatment.

Artificial intelligence (AI) has emerged as a promising toolkit in cardiology for the prediction of various cardiomyopathies feasible at large scale.^6^ Using electrocardiograms (ECGs), predictive AI models have been built for various cardiomyopathies^7^, ^8^, ^9^ including HCM.^10^^,^^11^ A major limitation of current studies is their reliance on the “ground truth” mostly being based on the screening of International Classification of Diseases (ICD) codes and/or echocardiography findings instead of the diagnostic certainty of cardiac magnetic resonance imaging (cMRI), the contemporary diagnostic gold standard. In this study, we present the performance of an AI algorithm that overcomes such limitations by using a cMRI-validated and physician-verified HCM diagnosis (Central Illustration).

**Central Illustration:**
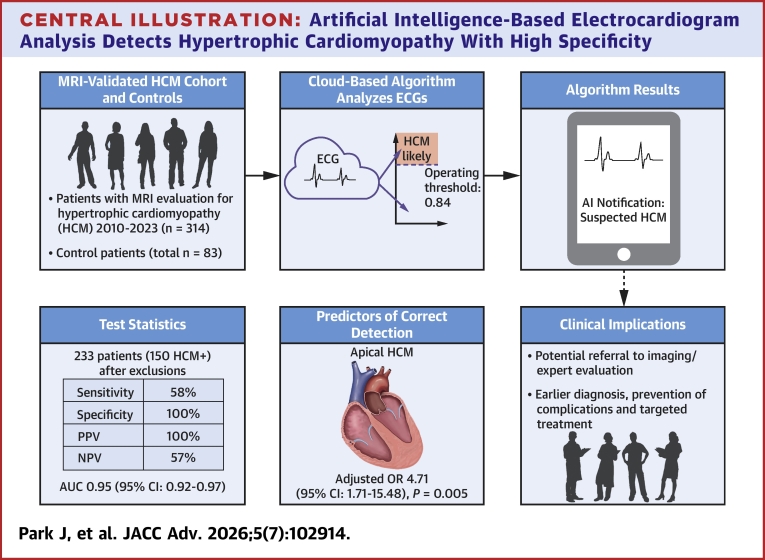
**Artificial Intelligence-Based Electrocardiogram Analysis Detects Hypertrophic Cardiomyopathy With High Specificity** Top panel: Data utilization pipeline used in current study and proposed for potential future screening workflow. 12-lead standard electrocardiogram data were analyzed by a cloud-based artificial intelligence algorithm which detects hypertrophic cardiomyopathy using a probability threshold-based classification. Bottom panel: Test characteristics of artificial intelligence-electrocardiogram algorithm for correct hypertrophic cardiomyopathy detection and significant predictors of correct detection. Abbreviations as in Figures 1 and 3.

## Methods

### Patient selection and confirmation of diagnosis

**Figure 1:**
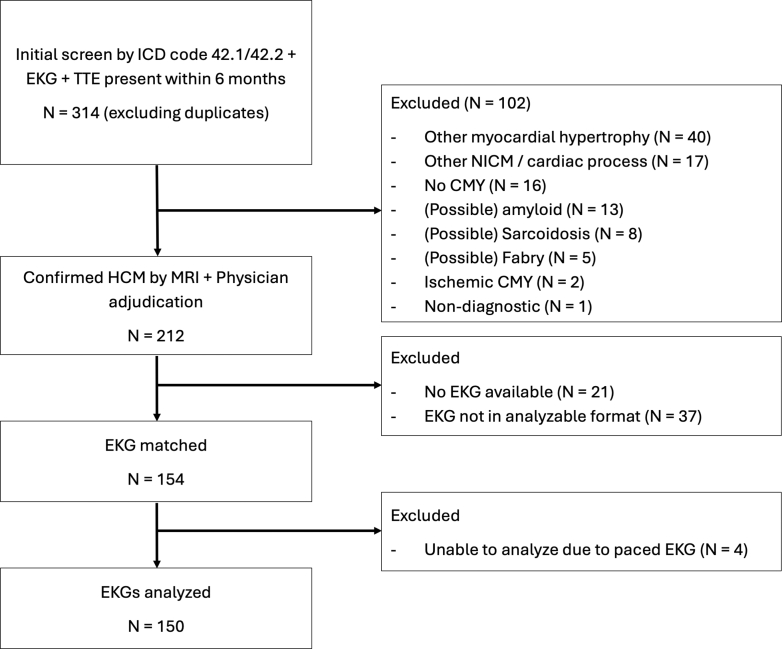
**Patient Selection Flowchart** CD = International Classification of Diseases; CMY = cardiomyopathy; ECG = electrocardiogram; HCM = hypertrophic cardiomyopathy; MRI = magnetic resonance imaging; NICM = non-ischemic cardiomyopathy; TTE = transthoracic echocardiogram.

We identified 314 consecutive adult patients at Cedars-Sinai Medical Center from 2010 to 2023 who underwent cMRI for evaluation of HCM and were identified by an electronic health record screen using ICD-10 codes 42.1 and 42.2 (Figure 1). For inclusion in the analysis, study patients required a positive diagnosis of HCM by cMRI that was confirmed by physician review of the cMRI report and electronic health record, according to guideline-based definitions for HCM using a maximal wall thickness (MWT) ≥15 mm (or ≥13 mm with a family history or genetic HCM diagnosis in absence of a hemodynamic explanation of the hypertrophy^12^). Additionally, the presence of an ECG that was analyzable by the AI algorithm was required. A total of 150 study patients met these criteria and were included.

Among patients in our health system without an ICD-10 code for HCM, control patients were identified after individually adjudicating each MRI report and excluding those with evidence of any features suggestive of myocardial and pericardial abnormalities (eg, pericardial inflammation or scarring, myocarditis, presence of scarring, and abnormal septal wall thickness). We identified 75 patients without any evidence of cardiomyopathy on cMRI (20 initial age-matched patients from 2010 to 2023 and an additional 55 patients from 2025 to enrich the control group for comparisons, per peer-review recommendations). Among the initial HCM-suspected group by ICD-10 code, we further identified 8 patients in whom there was a suspicion or family history of HCM but without any evidence of cardiomyopathy on cMRI—for a total of 83 control patients. The Cedars-Sinai Institutional Review Board approved this retrospective study with the requirement for individual informed consent waived (IRB ID STUDY00002872).

### Patient, clinical, and imaging characteristics

Demographic data and clinical characteristics were abstracted from patient charts. Echocardiographic variables included left ventricular ejection fraction (LVEF) and peak resting left ventricular outflow tract (LVOT) gradient. Cardiac MRI variables were comprised of LVEF (by cMRI), MWT, presence or absence of late gadolinium enhancement (LGE), amount of LGE in %, and hypertrophy type (septal, apical, concentric, and other). The presence or absence of cMRI high-risk markers for sudden cardiac death (left ventricular wall thickness ≥30 mm, delayed contrast enhancement ≥15% of left ventricle, and presence of apical aneurysm) from the report was recorded if available. The presence of at least one cMRI high-risk marker resulted in the classification of a patient as “high clinical risk.”

### ECG eligibility and analysis

Patients aged ≥18 years without pacemaker underwent analysis with Viz HCM (Viz.ai, Inc), a proprietary, food and drug administration-approved (DEN230003) AI algorithm trained on 831,329 total ECGs from 301,106 total patients (4,470 positive and 298,394 negative for HCM), split into sets comprising 80% and 20% of the data for respective training and validation. Details of the ECG signal processing and classifier algorithm have recently been published.^13^^,^^14^ Briefly, the primary image-based ECG data (from Cedars-Sinai database, General Electric MUSE) are converted into tensor inputs for a series of convolutional neural networks which generates a probability score for HCM ranging from 0 (no HCM) to 1 (100% probability of HCM). The algorithm operating point or threshold to classify an ECG as positive for HCM was 0.84 in this study and another recent publication,^13^ which was used to achieve high specificity with the goal to minimize false-positive outputs in a low-prevalence population.

### Statistical analysis

The statistical analyses were performed using R version 4.4.2. Continuous data were presented as median (IQR), and categorical variables were presented as absolute and relative frequencies. Missing data were not imputed. Comparisons between groups were performed with the use of Mann-Whitney *U* test for continuous variables and chi-square or Fisher exact test as appropriate for categorical variables. To determine the test performance of the AI algorithm, the area under the curve (AUC) of the receiver-operating characteristic curve was determined using the physician-adjudicated HCM status on cMRI of the study patients as the ground truth. If there were multiple ECGs on the date closest to the ground-truth MRI date, then the algorithm-defined HCM status was determined by the median value of their probability scores. Univariable and multivariable logistic regression was performed to determine the association between predictors of correct identification of HCM by the AI algorithm and selected patient demographic, clinical, and imaging variables. The multivariable logistic regression model included age, sex, race, MWT, and hypertrophy subtype. The individual predictor *P* values were determined by the Wald test. CIs for the AUC were calculated using bootstrapping with 2000 intervals, and CIs for sensitivity, specificity, positive predictive value, and negative predictive value were calculated using the Wilson method. In the time-based analysis of algorithm results, the median value of multiple ECGs within a time range was used for each patient. The AI probability scores in each cohort were not normally distributed (confirmed by Q-Q plots); a logit transformation with a 0.001 offset normalized the scores, and the resulting transformation was analyzed by 2-way analysis of variance to evaluate the effects of cohort and time. For all analyses, a 2-tailed *P* value of <0.05 was required to reject the null hypothesis.

## Results

### Patient characteristics

Among 233 total patients (150 with MRI-confirmed, physician-adjudicated diagnosis of HCM, and 83 control patients), age was significantly higher among HCM patients (63 vs 49 years, *P* < 0.001), as shown in Table 1. As expected, HCM patients had a greater median LVEF by cMRI (67% vs 61%, *P* < 0.001) and by transthoracic echocardiogram (67% vs 60%, *P* < 0.001), greater MWT by cMRI (18.0 mm vs 10.0 mm, *P* < 0.001), and presence of LGE compared to controls (77% vs 0%, *P* < 0.001). Among HCM patients, 21 of 93 (23%) had echocardiographic LVOT obstruction (defined by an LVOT gradient ≥30 mm Hg).

**Table 1:** Patient Characteristics by HCM Status According to Physician-Adjudicated Cardiac MRI

	cMRI Positive (n = 150)	cMRI Negative (n = 83)	*P* Value
Demographic information			
Age, y, median (Q1-Q3)	63 (49-69)	49 (32-64)	<0.001
Male, N (%)	96 (64%)	49 (59%)	0.268
Race			0.268
Asian	18 (12%)	12 (16%)	
Black	28 (18%)	7 (9%)	
White	92 (62%)	53 (69%)	
Other	10 (7%)	5 (7%)	
Unknown	2	6	
Hispanic ethnicity	16 (11%)	6 (8%)	0.419
Unknown	2	3	
Clinical characteristics			
HCM-causing gene mutation, n (%)	24 (49%)	1 (1%)	<0.001
Unknown	101	0	
Body mass index in kg/m^2^, (median, Q1-Q3)	27.3 (23.8-30.5)	25.8 (23.0-30.2)	0.153
Unknown	1	7	
Hypertension, n (%)	119 (80%)	32 (39%)	<0.001
Unknown	1	1	
Hyperlipidemia, n (%)	111 (74%)	18 (29%)	<0.001
Unknown	0	20	
History of smoking, n (%)	43 (29%)	19 (25%)	0.523
Unknown	0	6	
Treatments			
Cardiac myosin inhibitor, n (%)	17 (11%)	0	0.001
Beta-blocker, n (%)	106 (71%)	28 (34%)	<0.001
Unknown	1	0	
Calcium-channel blocker, n (%)	19 (13%)	3 (4%)	0.021
Unknown	3	0	
Disopyramide, n (%)	8 (6%)	0	0.053
Unknown	4	0	
Septal reduction therapy, n (%)	21 (14%)	0	<0.001
Unknown	2	0	
cMRI data			
LVEF in %, median (Q1-Q3)	67 (60-72)	61 (56-64)	<0.001
Unknown	2	1	
Maximal wall thickness in mm, median (Q1-Q3)	18.0 (16.0-21.0)	10.0 (9.0-11.0)	<0.001
Unknown	2	20	
Presence of LGE, n (%)	115 (77%)	0	<0.001
Unknown	1	0	
Amount of LGE in %, median (Q1-Q3)	7 (0-14)	0 (0)	<0.001
Unknown	66	0	
Echo data			
LVEF in %, median (Q1-Q3)	67 (62-74)	60 (59-66)	<0.001
Unknown	42	18	
Peak resting LVOT gradient in mm Hg, median (Q1-Q3)	8 (5-26)	4 (3-5)	<0.001
Unknown	57	38	
LVOT gradient >30 mm Hg, n (%)	21 (23%)	0	<0.001
Unknown	57	38	

cMRI = cardiac magnetic resonance imaging; HCM = hypertrophic cardiomyopathy; ICD = International Classification of Diseases; LVEF = left ventricular ejection fraction; LVOT = left ventricular outflow tract; MRI = magnetic resonance imaging.

### Test characteristics

**Figure 2:**
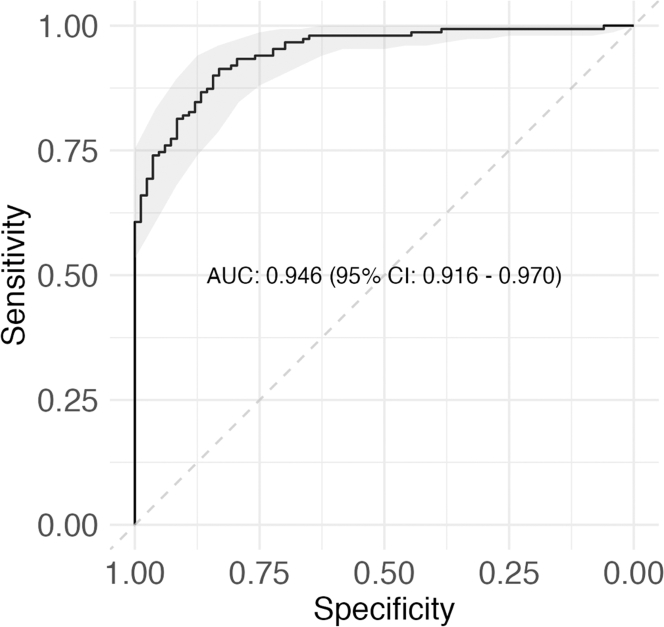
**The Receiver-Operating Characteristic Curve for the Electrocardiogram Analysis Algorithm** The gray shading indicates the 95% CI of the sensitivity at each specificity level. AUC = area under the curve.

A total of 87 out of 150 patients with HCM were correctly identified by the algorithm, resulting in a sensitivity of 58% (95% CI: 50.0% -65.6%). All 83 controls patients were correctly identified as not having HCM, thus yielding a specificity of 100% (95% CI: 95.6%-100%). There were no false positive flags by the algorithm, resulting in a positive predictive value of 100% (95% CI: 95.8%-100%). The complete test characteristics with receiver-operating characteristic curve are shown in Figure 2 and Central Illustration. The model had an AUC of 0.946 (95% CI: 0.916-0.970).

### Comparison of characteristics of correctly identified vs falsely undetected patients

The characteristics of the patients who were correctly identified as positive (“true positive”) were compared to the group that was missed (“false negative”). Among demographic and clinical characteristics, only male sex (correct detection in 72% vs 52%, *P* = 0.012) and lower age (correct detection, mean age 60 vs 66 years, *P* = 0.03) showed a statistically significant difference, while there were no significant differences in race, Hispanic ethnicity, hypertension, or body mass index (Table 2). Of the imaging variables, there was a statistically significant difference in hypertrophy type (*P* = 0.007); notably, the rate of the apical subtype was much higher in the correctly identified group (28% vs 8%). There were no significant differences in other imaging variables: LVEF, MWT, or presence and amount of LGE. Furthermore, no differences were seen in the rate of high clinical risk for sudden cardiac death, treatment status with cardiac myosin inhibitors, or HCM positivity by genetic testing.

**Table 2:** Patient Characteristics by True Positive and False Negative Algorithm Designation

	True Positive (n = 87)	False Negative (n = 63)	*P* Value
Demographic information			
Age, median (Q1-Q3)	60 (48-67)	66 (50-71)	0.030
Male, n (%)	63 (72%)	33 (52%)	0.012
Race, n (%)			0.876
Asian	10 (12%)	8 (13%)	
Black	17 (20%)	11 (18%)	
White	52 (60%)	40 (65%)	
Other	7 (8%)	3 (5%)	
Unknown	1	1	
Hispanic ethnicity	7 (8%)	9 (15%)	0.218
Unknown	1	1	
Clinical characteristics			
HCM-causing gene mutation, n (%)	12 (46%)	12 (52%)	0.674
Unknown	61	40	
Body mass index in kg/m^2^, median (Q1-Q3)	27.3 (24.1-31.0)	26.9 (23.6-30.0)	0.699
Unknown	1	0	
Hypertension, n (%)	70 (80%)	49 (79%)	0.830
Unknown	0	1	
Hyperlipidemia, n (%)	65 (75%)	46 (73%)	0.815
History of smoking, n (%)	27 (31%)	16 (25%)	0.451
Treatment with cardiac myosin inhibitor	9 (10%)	8 (13%)	0.654
cMRI data			
LVEF in %, median (Q1-Q3)	67 (60-72)	66 (58-71)	0.398
Unknown	2	0	
Maximal wall thickness in mm, median (Q1-Q3)	19.0 (16.0-22.0)	18.0 (16.0-20.0)	0.061
Unknown	0	2	
Presence of LGE, n (%)	69 (80%)	46 (73%)	0.300
Unknown	1	0	
Amount of LGE in %, median (Q1-Q3)	8 (1-14)	6 (0-11)	0.220
Unknown	38	28	
High clinical risk, n (%)	18 (23%)	13 (24%)	0.908
Unknown	8	8	
Hypertrophy type			0.007
Septal predominance	59 (68%)	55 (87%)	
Apical predominance	24 (28%)	5 (8%)	
Concentric predominance	3 (3%)	3 (5%)	
Other	1 (1%)	0	
Echo data			
LVEF in %, median (Q1-Q3)	68 (64-75)	65 (61-70)	0.055
Unknown	23	19	
Peak resting LVOT gradient in mm Hg, median (Q1-Q3)	10 (5-35)	7 (5-14)	0.333
Unknown	32	25	

Abbreviations as in Table 1.

The relationship between correct HCM identification and select demographic and clinical variables was further assessed by univariable and multivariable logistic regression models (Table 3). In univariable logistic regression, male sex, MRI MWT, and apical hypertrophy subtype of HCM were associated with correct HCM identification by the algorithm. In a multivariable-adjusted logistic regression model of age, sex, race, and selected cMRI variables used in clinical practice (MWT and hypertrophy subtype), apical hypertrophy subtype of HCM (adjusted OR: 4.709; 95% CI: 1.708-15.48; *P* = 0.005) was the only statistically significant predictor.

**Table 3:** Logistic Regression Analysis for Predictors of Correct HCM Identification by the Algorithm

	Univariable OR (95% CI)	*P* Value	Multivariable OR (95% CI)	*P* Value
Age	0.981 (0.957-1.004)	0.107	0.987 (0.961-1.012)	0.308
Male	2.386 (1.212-4.763)	0.012	1.799 (0.845-3.835)	0.127
Race (reference: White)				
Asian	0.962 (0.348-2.730)	0.940	1.250 (0.416-3.866)	0.692
Black	1.189 (0.506-2.882)	0.695	0.885 (0.332-2.382)	0.807
Other	1.795 (0.467-8.730)	0.417	1.676 (0.397-8.693)	0.499
MRI maximal wall thickness	1.098 (1.012-1.202)	0.033	1.091 (0.997-1.205)	0.069
Hypertrophy subtype (reference: septal)				
Concentric	0.932 (0.166-5.221)	0.933	0.970 (0.165-5.705)	0.972
Apical	4.475 (1.713-14.02)	0.004	4.709 (1.708-15.48)	0.005

Abbreviations as in Table 1.

Independent variable: Correct identification of HCM.

Dependent variables: age, sex, race, MRI maximal wall thickness, hypertrophy subtype.

### Exploratory analysis for time-based diagnostic yield and lead time to positive cardiac MRI

**Figure 3:**
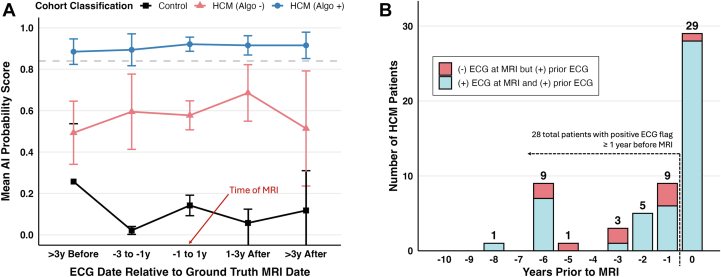
**Classification of Electrocardiograms Overtime Relative to Ground-Truth Magnetic** Resonance Imaging **Date** (A) The mean artificial intelligence probability score with 95% CIs by one of 5 time bins and 3 cohorts: control (hypertrophic cardiomyopathy negative), hypertrophic cardiomyopathy positive and algorithm negative (hypertrophic cardiomyopathy Algo-, false negative), and hypertrophic cardiomyopathy positive and algorithm positive (hypertrophic cardiomyopathy Algo +, true positive). (B) Patients with electrocardiograms flagged as hypertrophic cardiomyopathy-positive prior to ground-truth magnetic resonance imaging. Each bar represents the number of hypertrophic cardiomyopathy-suspected patients in the year leading up to the indicated interval. A total of 28 patients were flagged hypertrophic cardiomyopathy-positive by artificial intelligence-electrocardiogram at least 1 year prior to the magnetic resonance imaging date. Of these 28 patients, 9 positive flags (6 positive and 3 negative by electrocardiogram closest to magnetic resonance imaging) were made by artificial intelligence-electrocardiogram prior to establishing a clinical diagnosis with a median lead-time of 2.6 (IQR: 1.1-5.3) years AI = artificial intelligence; ECG = electrocardiogram; other abbreviations as in Figure 1.

To determine whether earlier ECG-based detection of HCM is possible prior to a truly positive MRI, all available ECGs surrounding an HCM patient’s cMRI study date were analyzed by the algorithm. A total of 132 patients out of 233 patients had multiple ECGs at different time points (before and after) the cMRI, 92/150 (61%) among HCM-confirmed patients and 30/83 (36%) among control patients, yielding a total of 550 HCM and 154 control ECGs. The algorithm demonstrated consistent HCM classification over multiple years before and after cMRI (Figure 3A). The mean AI probability scores were significantly different over time between true positive, false negative, and control patients (2-way analysis of variance, F(2,280) = 314, *P* < 0.001).

A total of 57 HCM-positive patients by ground-truth had an algorithm-positive ECG before cMRI; 28 (49%) of these patients (20 true positives and 8 false negatives as classified by the ECG closest to cMRI date), had an ECG that was flagged positive by the algorithm at least 1 year prior to the cMRI (Figure 3B). The medical record of all 28 “early” flags was reviewed, and 9 patients (or 32%) did not have an HCM diagnosis at the time of earliest positive flag on ECG. Notably, 4 out of those 9 patients showed imaging findings indicating high clinical risk at the time of cMRI. The median (interquartile) “lead-time” from AI-positive ECG preceding diagnosis to cMRI was 2.6 (1.1-5.3) years.

## Discussion

In this study, we evaluated the performance of an AI-based ECG analysis algorithm to correctly identify HCM patients in a rigorously validated cohort with a cMRI-confirmed and physician-adjudicated HCM diagnosis—a key strength that sets this investigation apart from prior studies. Our work suggests that the algorithm can achieve identification with high specificity, which is consistent with another validation involving a much lower prevalence of HCM in the cohort.^11^ This study further sheds light on clinical and imaging variables that are associated with an improved detection of HCM by the algorithm.

### Ground truth validation by cardiac MRI

A key strength of this study lies in its use of cMRI as the ground truth for validating the AI algorithm’s performance. Previous AI models for HCM detection have often relied on less robust diagnostic criteria, such as ICD code search; however, there is substantial evidence that in HCM, misclassification in registry databases by ICD code occurs in up to one-third of patients with positive predictive values as low as 68%^15^^,^^16^ and common confounders include hypertension and aortic stenosis-related heart disease.^17^ This outcome is similar to the initial exclusion rate of 39% (Figure 1) in our study, with 40 out of 102 patients having hypertrophy due to a diagnosis other than HCM even after screening for ICD codes I42.1/42.2 which are explicitly reserved for HCM.

Cardiac MRI has increasingly been acknowledged to have superior diagnostic performance for the detection and characterization of HCM compared to echocardiography, especially in ambiguous cases due to its high spatial resolution, high blood-tissue contrast, and improved abilities for tissue characterization.^12^^,^^18^^,^^19^ In a meta-analysis by Queiroz et al that investigated the diagnostic performance of ECG analysis tools to detect HCM, a majority of studies applied a diagnosis definition of HCM based on health record review and echocardiographic criteria.^20^ Given evidence suggesting that even referral centers reclassify an incorrect diagnosis of HCM after incorporating cMRI in their diagnostic process,^21^ the former approach might be insufficient—although practical feasibility of individual adjudication of MRI reports in large-scale studies needs to be considered. In contrast, our validation cohort benefits from the high confidence and fidelity in the ground truth of the HCM diagnosis by incorporating both physician review and cMRI for each patient.

### Sensitivity and specificity considerations

The performance of the algorithm presented in this study shows a sensitivity of 58%, a specificity of 100%, and a positive predictive value of 100%. A recent meta-analysis investigating the diagnostic performance of convolutional neural networks ECGs for HCM showed a pooled sensitivity and a specificity of 89% and 88%, respectively.^20^ Our observation of a comparatively lower sensitivity while maintaining high specificity is likely the result of the study algorithm’s deliberate design to adjust the operating point to achieve very high specificity.^22^ This consideration is particularly critical when screening for a disease with a low prevalence, such as HCM, with an estimate of 0.2% to 1.4% in the general population.^1^^,^^2^ By increasing test sensitivity at the cost of reducing specificity, at large scale, even a small increase of false positives could easily overwhelm clinical capacity (as shown in recent simulations^23^), lead to unnecessary downstream testing like cMRI, specialist consultations, and hence significantly reduce the cost-effectiveness of a potential screening mechanism—although the balance might change if utilized in HCM-enriched populations.^24^^,^^25^ An approach highlighting high sensitivity was recently demonstrated through work by Swain et al who showed that in a small cohort of survivors of sudden cardiac arrest, 96% of patients had a high probability for HCM by AI detection. The utility of such approach, however, is questionable, as such high-risk patients almost certainly will get a comprehensive workup for the cause of their arrest, including advanced imaging to rule out HCM.^26^ In contrast, a highly specific algorithm could be applied to large patient populations within a health system, with minimal *unnecessary* diagnostic workup or triggering of patient concerns. Thus, in a general population (eg, all-comers in clinic), the authors envision a reliably specific “rule-in” than a highly sensitive “rule-out” mechanism for HCM and consider it paramount to appropriately optimize the test characteristics based on its potential downstream effects in a real-world setting.

### Variables associated with correct identification

The study further investigated factors associated with the correct identification of HCM by the AI algorithm. Our analysis suggests that a greater MWT and apical subtype are associated with increased odds of correctly identifying HCM by AI-ECG analysis. This outcome could potentially be explained by a higher disease burden or progression that resulted in increased phenotypic discrimination detectable via ECG. The apical subtype of HCM is more commonly associated with prominent ECG changes (“Yamaguchi syndrome”) which in recent literature have been shown to be detectable with high levels of accuracy by AI.^27^^,^^28^ Work by Siontis et al suggests that AI-assisted ECG analysis could potentially differentiate between various disease stages of HCM—however, conclusive data are limited to date.^29^ Notably, while not available in all study patients, our analysis of high-risk features including MWT, late gadolinium enhancement, and presence of apical aneurysm—which presumably are clinical surrogates of disease progression and burden—did not demonstrate an increased correct detection rate of HCM. These contrasting results show the challenge that phenotypical differences do not always translate into detectable ECG changes. HCM disease progression involves a complex interplay of myocardial mass, geometry, fiber arrangement, and fibrosis which affects electrical activation patterns in different ways. As a result, ECG signals may not change in a consistent direction with advanced disease (eg, increasing fibrosis causing lower ECG voltages), as recent computational models have discussed.^30^ Our study population notably shows statistically significant differences in mean AI score with the lowest probability in controls, and a gradually higher probability in false negative and true positive patients; however, a feature-level analysis of the ECGs (eg, via GradCAM) could not be performed. Identifying and characterizing these progressive and fluctuating changes should be investigated further to enhance the accuracy of future algorithms.

### Clinical utility in addressing the diagnostic gap

Large-scale AI-assisted ECG analysis with high specificity holds significant potential to improve detection of HCM and reduce the diagnostic delay in the general population.^31^ Our study simulated such an approach by reviewing all available ECGs of AI-flagged patients to determine the proportion of patients that could have been alerted for HCM evaluation and diagnosis prior to their actual diagnosis made by a clinician. This AI-based screening could detect undiagnosed HCM at high rates (9 out of 28 – or 32%) in patients with ECGs available 1 year prior to MRI, including those who were later found to have high clinical risk for sudden cardiac death at the time of cMRI. The median lead-time prior to diagnosis was 2.6 years; in clinical practice, such patients could benefit from earlier diagnostic confirmation, HCM-specific treatment, and prevention of sudden cardiac death.^30^

The potential for the algorithm to flag early HCM suspicion on ECG prior to ground-truth cMRI and diagnosis is consistent with a prior recent study^31^ and our observed separation in the mean AI probability score between HCM-confirmed patients and controls across all time periods of available ECGs relative to ground-truth MRI. An important consideration when interpreting any given AI output is the real-world alignment to the ground truth since an AI probability score does not necessarily match the real-world positive predictive value. To improve such real-world alignment and overall clinical applicability of an AI output, various approaches including federated learning,^32^ the incorporation of specific clinical variables such as a risk score^33^ and “Platt scaling” are currently being developed.^13^

While not routinely implemented in the real-world setting yet, ECG tools can already be seamlessly integrated into clinical workflows and electronic health records in various clinical settings including primary care and population health screening programs, as well as emergency and inpatient settings.^34^ A reliably predictive ECG tool with minimal false positive flags could lead to earlier diagnosis, potentially mitigating the higher morbidity, mortality, and economic burden associated with unnecessary downstream testing. However, whether the incorporation of AI-assisted tools in routine cardiovascular care truly results in improved long-term health outcomes needs to be further evaluated.^22^^,^^23^^,^^35^^,^^36^

### Study Limitations

There are several limitations of this study that warrant consideration. The single-center analysis and limited sample size of the control group may restrict the generalizability of the findings. Due to the relatively specific control group, the algorithm was not challenged to discriminate between HCM phenocopies that can be encountered in a real-world, general population screening scenario—hence, the true specificity might be lower. Future investigations would require further validation against a broader, HCM-negative population. However, such studies are difficult to perform due to the overlap with other conditions including hypertensive heart disease and genotype-positive but phenotype-negative HCM. Therefore, the goal of our study was to evaluate only pure comparison groups. Furthermore, the retrospective nature of the study inherently introduces potential selection biases as patients identified via ICD-10 codes for HCM and evaluation by cMRI might represent a cohort with more phenotypically advanced disease than all-comers or undiagnosed HCM patients in the general population. Finally, the algorithm was intentionally optimized for high specificity which may limit detection of all screened HCM cases. However, the authors believe that increasing the sensitivity of a 12-lead ECG from essentially zero in a real-world clinical setting (ie, cardiologists typically do not diagnose HCM solely by ECGs) to 58% is impactful.

## Conclusions

In this single-center retrospective analysis, an ECG-based AI algorithm demonstrated highly specific identification of HCM confirmed by cardiac MRI. Applying an ECG algorithm for screening purposes could help identify previously unrecognized HCM patients who may benefit from further diagnostic workup and therapies.

Perspectives **MEDICAL KNOWLEDGE:** An AI-ECG algorithm can identify HCM with high specificity and has the potential for large-scale screening that mitigates unnecessary downstream testing. Screening efforts utilizing AI may therefore improve current underdiagnosis and clinical outcomes of affected patients.

**TRANSLATIONAL OUTLOOK:** Future work should focus on integration with longitudinal clinical data, refinement across HCM phenotypes, and prospective studies to determine whether AI-guided screening leads to earlier diagnosis and improved clinical outcomes.

## Funding support and author disclosures

This work was supported by the Smidt Family Foundation. J.P. drafted the manuscript. F.R. conceived and supervised the research. J.P., C.J.L., and F.R. made substantial edits to the manuscript. J.P., J.K., K.R.R., A.K., S.M., K.K., and F.R. collected the data. C.J.L. analyzed all data. All authors reviewed and approved the final manuscript. Drs Love, Mr. Meshesha and Ms. Kon disclose employment with Viz.ai. All other authors have reported that they have no relationships relevant to the contents of this paper to disclose.
